# Effects of* Juniperus phoenicea* Hydroalcoholic Extract on Inflammatory Mediators and Oxidative Stress Markers in Carrageenan-Induced Paw Oedema in Mice

**DOI:** 10.1155/2018/3785487

**Published:** 2018-07-09

**Authors:** Karama Zouari Bouassida, Samar Makni, Amina Tounsi, Lobna Jlaiel, Mohamed Trigui, Slim Tounsi

**Affiliations:** ^1^Biopesticides Laboratory (LPIP), Center of Biotechnology of Sfax, University of Sfax, P.O. Box 1177, 3018 Sfax, Tunisia; ^2^Analysis Department, Center of Biotechnology of Sfax, University of Sfax, P.O. Box 1177, 3018 Sfax, Tunisia

## Abstract

*Juniperus phoenicea *(*J. phoenicea*) is a wild tree belonging to the Cupressaceae family, commonly used for the treatment of several disorders. This study aimed to evaluate the potential protective effects of* J. phoenicea* hydroethanolic extract (EtOH-H_2_OE) against oxidation, acute inflammation, and pain in mice models. For the purpose, chemical compounds of* J. phoenicea* EtOH-H_2_OE were also analyzed by GC-MS. The* J. phoenicea* EtOH-H_2_OE showed a potent antioxidant activity* in vitro*, thanks to its richness in phenolic and flavonoid compounds. Mice treated with EtOH-H_2_OE (100 mg/kg BW) showed reduced paw oedema formation and decreased malondialdehyde (MDA) content. The evaluation of antioxidant enzyme activities in paw oedema tissue after five hours of carrageenan induction showed a significant increase (*P* < 0.05). Inflammatory biomarkers explorations of* J. phoenicea* EtOH-H_2_OE-treated mice showed a restoration of the studied parameters to near-normal values. Furthermore, EtOH-H_2_OE of* J. phoenicea *produced a significant reduction of the number of abdominal writhes (*P* < 0.05) in a dose-dependent way. Phytochemical analysis of the* J. phoenicea* EtOH-H_2_OE by GC-MS showed the presence of hexadecanoic and stearic acids known as anti-inflammatory and analgesic compounds. Our investigation provided evidence that* J. phoenicea* EtOH-H_2_OE can effectively reduce the inflammation and pain in mice models.

## 1. Introduction

Medicinal plants have been used in traditional health care systems since prehistoric civilizations. World Health Organization (WHO) estimates that about 85% of the worldwide traditional knowledge involves the use of plant extracts [1]. Therefore, treatment of various diseases has been carried out with medicinal plants for many years. These plants are an endless source of pharmaceutical and therapeutic products, thanks to their richness in bioactive molecules that have medicinal properties. The antioxidant compounds derived from plant extracts could be a solution to overcome diseases, caused by oxidative stress, such as inflammation [2].

Oxidative stress is the result of an imbalance between the reactive oxygen species (ROS) levels production during metabolism and their removal by the antioxidant defense systems [3]. Excessive production of ROS in tissues may contribute to cell damage by altering cellular molecules, namely, proteins, lipids, and DNA/RNA [4, 5]. Furthermore, it was indicated that ROS excess initiates the inflammation process by stimulating the induction of proinflammatory cytokines, chemokines, and pro-inflammatory transcription factors causing tissue injury [6]. Inflammation represents a rapid yet coordinated set of events that enable tissues to respond to injuries or infections [7]. It is a natural defense mechanism that involves the recruitment of immune cells and the overproduction of inflammatory cytokines into the tissues in response to the ROS release [8]. To avoid the ROS harmful effects, human cells have developed a sophisticated antioxidant defense system that consists of an enzymes system involved in the conversion of ROS to less reactive molecules such as O_2_ and water [9]. Superoxide dismutase (SOD), catalase (CAT), and glutathione peroxidase (GPx) are the main endogenous enzymes involved in protecting aerobic cells from the noxious effects of ROS. They give protection by directly scavenging superoxide radicals and hydrogen peroxide, converting them into stables species [10]. Cell damage induced by an excessive production of ROS depends on the potential of the cellular antioxidant enzymes to neutralize them [11]. Studies documented that, in addition to the enzymatic/nonenzymatic cellular antioxidant defense system's protective effects, natural products having antioxidant activities may lead to delaying the oxidative stress and inflammatory tissue damage by enhancing the cell/tissues defenses [12].

The search for natural products with antioxidant activities has increased enormously over the last decades. Plants would be a promising alternative to reduce oxidative damage, thanks to their secondary metabolites richness such as polyphenols, flavonoids, tannins, terpenoids, and anthraquinones [13]. It was reported that plant natural compounds are able to interact with ROS and thus terminate the chain reaction before the cell vital molecules were seriously damaged [14].

*Juniperus phoenicea is* a wild tree belonging to the Cupressaceae family and is popularly known as “Arâar” in Tunisia.* Juniperus* species have been widely used in the traditional medicines against various infectious and inflammatory diseases such as cold, diarrhea, fungal infections, hemorrhoids, leucorrhea, diabetes, and wounds [15]. In Tunisian folk medicine, the* J. phoenicea *leaf decoction was frequently employed to regulate menstruation and relieve the pain of menstrual cramps [16]. The mixture of berries and leaves of this plant was used traditionally as antidiabetic remedies [17]. Previous studies on the biological activities of* J. phoenicea *have mainly focused on the essential oils. Although the plant is claimed to be used for its anti-inflammatory and analgesic effects by Tunisian traditional healers, no published work so far investigates the use of* J. phoenicea *for this purpose.* J. phoenicea* has a long use record in the treatment of oedema in the Mediterranean subdivision [15].

This study aimed mainly to (1) explore the properties of* J. phoenicea *EtOH-H_2_OE in the management of inflammation and oxidation using carrageenan-induced paw oedema model, (2) evaluate the analgesic potential of* J. phoenicea *EtOH-H_2_OE using acetic acid-induced abdominal contraction, (3) identify the phytochemicals present in the extract by GC-MS, and (4) assess the relationship between the extract agents and the bioactivities.

## 2. Materials and Methods

### 2.1. Drugs and Reagents

2,2-Diphenyl-1-picrylhydrazyl (DPPH), butylated hydroxytoluene (BHT), linoleic acid, *β*-carotene, ascorbic acid, gallic acid, quercetin, Folin-Ciocalteu reagent, Lambda carrageenan, dexamethasone, and acetic acid were purchased from Sigma-Aldrich Company (Sigma Chemical Company, USA).

### 2.2. Plant Material

*J. phoenicea* fresh leaves were collected in January from Boulifa (El Kef, northwestern part of Tunisia: 36°07′25.7^″^N, 8°43′07.6^″^E). The plant sample was identified morphologically by Professor Mohamed Chaieb, the Department of Botany and Plant Ecology of the Faculty of Sciences (University of Sfax, Tunisia). A voucher specimen was kept at the Laboratory of Biopesticides of the Centre of Biotechnology of Sfax under the reference number LBPes J.P. 01.16.

### 2.3. Plant Extraction

The* J. phoenicea* air-dried leaves (105 g) were crushed into small parts with a blender and were sequentially extracted by hydroalcoholic maceration in ethanol-water (8:2, v/v) for 48 h under continuous shaking. The resulting extract (EtOH-H_2_OE) was filtered through a Whatman paper (pore size: 0.45 *μ*m, diameter: 47 mm) and then concentrated to afford 22.69 g which made the crude extract. EtOH-H_2_OE was kept at +4°C in the dark until further use.

### 2.4. Qualitative Analysis

The qualitative phytochemical tests were carried out according to Sofowora [18] and Harborne [19]. They were based on the visual observation of color change of EtOH-H_2_OE of* J. phoenicea*. The tested phytochemicals are phenolics, flavonoids, anthraquinones, glycosides, terpenoids, tannins, saponins, and alkaloids. The results are expressed as “+” for the presence and “-” for the absence of phytochemicals.

### 2.5. Gas Chromatography-Mass Spectrometry (GC-MS) Analysis

GC-MS analysis of EtOH-H_2_OE of* J. phoenicea* was performed with an Agilent 6890N Network GC System (Agilent Technologies). The system was equipped with an HP-5 MS column having 30 m × 0.25 mm i.d. × 0.25 *μ*m film as dimensions. The used system was coupled to a mass selective detector and the carrier gas was helium. The GC oven temperature started at 40°C and was held at 60°C for 2 min and was then programmed to rise from 60 to 325°C at a rate of 5°C/min. The injected extract temperature was set at 280°C.

The components identification was achieved by careful examination of fragmentation patterns and spectral data obtained from the Wiley and NIST libraries. This determination was carried out in duplicates.

### 2.6. Phenolic Contents and Antioxidant Activity

#### 2.6.1. Determination of Total Phenols

The total phenolic content was assayed using the Folin-Ciocalteu reagent and gallic acid as a standard [20]. The absorbance was measured at 760 nm and the results were expressed as mg of gallic acid equivalent per g (mg GAE/g). The assays were performed in triplicates.

#### 2.6.2. Determination of Total Flavonoids

The total flavonoid content in EtOH-H_2_OE was estimated by the aluminum chloride spectrophotometric method [21]. The mixture absorbance was read at 430 nm and the result was expressed as mg of quercetin equivalent per g dry weight (mg QE/g). Tests were performed in triplicates.

### 2.7. Determination of DPPH Radical Scavenging and *β*-Carotene Bleaching Capacity

The free radical scavenging capacity and the ability of the EtOH-H_2_OE to avoid *β*-carotene bleaching were determined using the methods described by Kirby and Schmidt [22] and Pratt [23], respectively. The ascorbic acid and the butylated hydroxytoluene (BHT) were taken as references.

### 2.8. Biological Activity Assays

#### 2.8.1. Animals

Mice (30–50 g) were obtained from the Central Pharmacy of Tunisia (SIPHAT, Tunisia). They were placed in cages at a controlled temperature of 22±2°C with free access to standard laboratory diet and drinking water. Experiments and protocols were carried out in accordance with the European Community guidelines (EEC directive of 1986; 86/609/EEC) for the care and use of laboratory animals in scientific research and approved by the Institutional Animal Ethics Committee (directive 2001-2133) issued by the University of Sfax, Tunisia.

#### 2.8.2. Acute Toxicity Study

The acute toxicity study was performed according to the World Health Organization recommendations (2000) with some modifications. Healthy mice (40 g) were randomly divided into four groups (*n* = 6). The control group (group I) received only a water solution, while groups II, III, and IV were treated with EtOH-H_2_OE of* J. phoenicea* at doses of 100, 200, and 400 mg/kg body weight, respectively. Following the fasting period, the graded doses of EtOH-H_2_OE of* J. Phoenicea* were administered orally by gavage. The animals were maintained on standard animal diet and water. All animals were daily observed for behavioral pattern, body weight, and physical appearance changes and checked for mortality during the 2-week observation period.

#### 2.8.3. Anti-Inflammatory Test: Carrageenan-Induced Paw Oedema

Anti-inflammatory activity of EtOH-H_2_OE of* J. phoenicea* was assessed according to the method described by Ravi et al. [24] with some modifications. Mice with a mean weight of 35 g were split into four groups (*n* = 6):

(i) Group I: mice were pretreated with 1 ml/kg of sterile saline solution by subplantar injection and had no inflammation (control group)

(ii) Group II: mice were inflamed by carrageenan injection 1% and did not receive any treatment (Carr)

(iii) Group III: mice received dexamethasone (100 mg/Kg BW) by intraperitoneal injection and were considered as standard (Carr + DEX)

(iiii) Group IV: mice were given 100 mg/kg BW of EtOH-H_2_OE of* J. phoenicea* (Carr + EtOH-H_2_OE of* J. phoenicea*) by intraperitoneal injection

Paw oedema was induced [25] by administration of 50 *μ*L of 1% w/v carrageenan solution (type IV, Sigma Chemical Company, USA) into subplantar tissues of the right hind paw of each animal after one hour of intraperitoneal administration of plant extract and drug.

The swelling of carrageenan injected paw was measured before and 1, 2, 3, 4, and 5 hours after the induction using a Digital Vernier Caliper [26]. The anti-inflammatory activity was calculated as percentage inhibition of oedema in the extract-treated animals under test in comparison to the carrageenan control group.

(1) $$\begin{array}{c} Percent\;\;inhibition=\left(\;1-\frac{PT}{P0}\;\right)\times 100, \end{array}$$

where PT represents the oedema volume of the drug-treated group and P0 is paw oedema of the carrageenan-treated group.

#### 2.8.4. Blood Sampling

Five hours after the carrageenan administration, the animals were anesthetized and the blood samples were collected in heparin tubes. Plasma samples were obtained after centrifugation (15 min at 4000 rpm) and they were kept in −20°C until further analysis. The pellet obtained after centrifugation on heparin will be used for the assays of the oxidative status markers.

#### 2.8.5. Exploration of Inflammatory Biomarkers

(*1) Hemogram Test.* The measurement of white blood cells and platelets is carried out with a hematology analyzer (KX21 hemogram).

(*2) Determination of C-Reactive Protein.* The serum C reactive protein levels were analyzed by an automatic analyzer “COBAS INTGRA 400.” The CRP is expressed in mg/L.

(*3) The Fibrinogen Assay.* The plasma fibrinogen assay was performed according to Clauss method [27] on the STA line analyzers using the STA fibrinogen reagent. The plasma samples were appropriately diluted with Owren-Koller buffer (pH 7.35). The fibrinogen concentrations are expressed in g/L.

(*4) Exploration of Oxidative Stress Parameters In Vivo. *The oxidative stress parameters were determined in skin tissues paw oedema. Homogenates were diluted (10%, w/v) in a Tris-buffered saline (pH 7.4) and centrifuged for 25 min (9000 rpm). The obtained supernatants were used for the determination of MDA as reported by Draper and Hadley [28].

The superoxide dismutase (SOD) content was colorimetrically assessed as reported by Beyer and Fridovich [29] using the inhibition of nitro blue tetrazolium (NBT). The catalase (CAT) activity was evaluated according to the method of Aebi [30] using H_2_O_2_ as substrate. The glutathione peroxidase (GPx) content was analyzed according to the method of Flohe and Günzler [31] and expressed as *μ*mol GSH/min/mg protein.

(*5) Histopathological Assessment of Skin Tissue.* Tissue specimen samples from subplantar muscles of all the studied groups were dissected five hours after carrageenan injection for histological examination. They were fixed in formalin solution (10%) and stained with hematoxylin-eosin. The sections were finally observed under a light microscope and photographed with an Olympus U-TU1X-2 camera.

### 2.9. Acetic Acid Writhing Test

The acetic acid-induced writhing test was performed as previously reported by Reza et al. [32] with some modifications. The experimental study designed six groups of 6 mice. Group I served as control and received distilled water. Groups II and III were treated with diclofenac sodium (standard analgesic drug) at 50 and 100 mg/kg, respectively. Groups IV, V, and VI were administered 50, 100, and 150 mg/kg of the EtOH-H_2_OE of* J. phoenicea*, respectively. After 30 minutes, acetic acid (1 mL of 1% w/v) was administered with intraperitoneal injection for writhing induction. The total number of abdominal constrictions was counted for each group of mice after 15 minutes of acetic acid injection for the period of 5 minutes.

The protection percentage against acetic acid was calculated using the following formula:

(2) $$\begin{array}{c} \%\;\;inhibition=\frac{number\;\;of\;\;writhes\;\;\left(control\right)-number\;\;of\;\;writhes\;\;\left(test\;\;group\right)}{number\;\;of\;\;writhes\;\;\left(control\right)}\times 100 \end{array}$$

### 2.10. Statistical Analysis

Data were expressed as mean values ± standard deviation (SD). A statistical significance comparison between groups was accomplished using the SPSS version 20.

The mean differences between the different groups were assessed by Duncan and Tukey's post hoc tests and compared using one-way analysis of variance (ANOVA). Differences were considered significant at* P* < 0.05.

## 3. Results

### 3.1. Phytochemical Analysis of Organic Extract

The phytochemical screening of hydroethanolic extract of* J. phoenicea* leaves revealed the presence of various secondary metabolites such as terpenoids, tannins, saponins, alkaloids, and anthraquinones, while gl**y**cosides were not detected in the tested extract (Table 1). Furthermore, the quantitative estimation of the total phenolic contents showed that the EtOH-H_2_OE contains the highest amount of phenolics (70.30 mg GAE/g) and total flavonoid contents (11.33 mg QE/g) (Table 2).

### 3.2. *J. phoenicea* EtOH-H_2_OE GC-MS Analysis

The identities of* J. phoenicea* constituents, their contents (%), and their retention times are listed in Table 3. The analysis of EtOH-H_2_OE of* J. phoenicea* leaves revealed the presence of 25 compounds belonging to different chemical classes. The compounds, hexadecanoic acid (7.41%), stearic acid (5.69%), eseroline (3.15%), and azelaic acid (2.45%), were the most abundant. This extract also contains some sugars including *β*-D-Glucopyranose (1.22%), L-Fructose (0.23%), and *α*.-D-Galactopyranoside (0.17%). Besides, EtOH-H_2_OE harbors some phenolic components including gallic, caffeic, and *ρ*-coumaric acids. EtOH-H_2_OE of* J. phoenicea* leaves revealed the presence of a considerable amount of saturated fatty acids (18.42 % of the total FAs).

### 3.3. Antioxidant Activity

As shown in Table 2, the DPPH radical scavenging activity was appreciated by the determination of the IC_50_ values. EtOH-H_2_OE of* J. phoenicea* exhibited a remarkable free radical scavenging activity with an IC_50_ value of 12.22 *μ*g/ml when compared to vitamin C (IC_50_ = 3.5*μ*g/l). In the *β*-carotene bleaching assay, the extract showed a potent activity (IC_50_ = 15 *μ*g/l) less than that of the BHT (5.1 *μ*g/l) taken as reference (Table 2).

A positive correlation between total phenolics content and antioxidant property (R^2^ = 0.972) was noted.

### 3.4. Acute Toxicity Study

EtOH-H_2_OE of* J. phoenicea* up to the dose of 400 mg/kg BW did not produce any signs of adverse reactions and no changes in the behavior of the treated animals up to 14 days following the extract administration. The treated animals did not display any abnormal signs such as food and water intake, convulsions, salivation, or diarrhea. No deaths or weight losses were recorded during the study. Therefore, EtOH-H_2_OE of* J. phoenicea* at 100 mg/Kg BW was used in the in* vivo* investigation of the anti-inflammatory activity.

### 3.5. Effects of* J. phoenicea* Leaf Extract on Carrageenan-Induced Paw Oedema

#### 3.5.1. Inhibitory Effect of the Extract on Paw Oedema* In Vivo*

**Figure 1:**
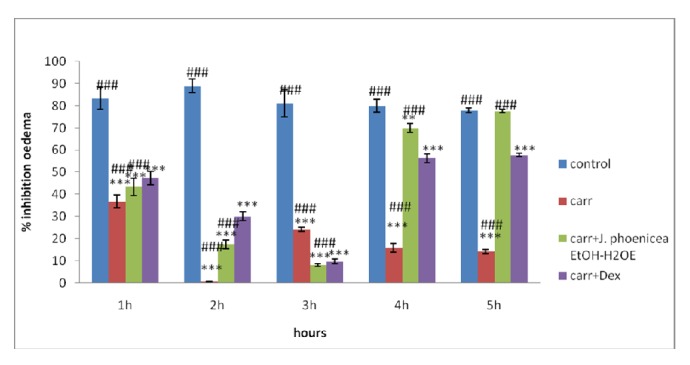
Percentage (%) of oedema inhibition data in all groups. Values represent mean ± SD (*n* = 6) in each group. *∗* represents* P *< 0.05, *∗∗* represents* P* < 0.01, and *∗∗∗* represents* P *< 0.001; # represents* P *< 0.05, ## represents* P* < 0.01, and ### represents* P *< 0.001; *∗* compared to control group and # compared to Carr + DEX. Control: physiological water, Carr: carrageenan, Carr + J: carrageenan +* J. phoenicea*, EtOH-H_2_OE, Carr + DEX: carrageenan + dexamethasone.

The comparative inhibitory effects following treatment with EtOH-H_2_OE of* J. phoenicea* and dexamethasone on carrageenan-induced paw oedema are presented in Table 4 and Figure 1. The subcutaneous administration of carrageenan generated an increase in the paw size in mice due to oedema. This paw oedema peaked after 3 hours of experiment, especially for the untreated group, thus indicating an acute paw inflammation. The experimental data of the treated group by EtOH-H_2_OE of* J. phoenicea* showed a significant decrease in the paw oedema size (*P* < 0.05), which was time-dependent and more important than the standard drug dexamethasone. Our findings revealed an inhibition of 77.5% of the paw oedema using the EtOH-H_2_OE compared to an inhibition of 57.89% using the standard drug after five hours. EtOH-H_2_OE of* J. phoenicea* (100 mg/kg BW) displayed a significantly (*P* < 0.05) higher anti-inflammatory capacity when compared to dexamethasone after 5 hours.

#### 3.5.2. Hematological, Biochemical, and Histopathological Examination

The macroscopic results were confirmed through hematological, biochemical, and histopathological explorations. The hematological parameters were assessed by monitoring the white blood cells and blood platelet levels. A significant increase in white blood cell count and blood platelets was shown in carrageenan inflamed group compared to the negative control group (Table 5). However, the inflamed animals, treated with the EtOH-H_2_OE of* J. phoenicea*, showed a significant decrease in the white blood cells and blood platelet counts and were close to those of control and dexamethasone-treated mice.

As shown in Table 4, the administration of carrageenan led to a significant enhancement in CRP level for the carrageenan group compared to the control animals. However, the CRP level significantly decreased (*P* < 0.05) in the groups treated with* J. phoenicea* (62.5%) and dexamethasone (50%) when compared to the carrageenan untreated group.

The fibrinogen rate decreased significantly (*P* < 0.05) in the groups of mice treated with the EtOH-H_2_OE of* J. phoenicea* (38.3%) and dexamethasone (37.42%) compared to the carrageenan untreated group (Table 5).

**Figure 2:**
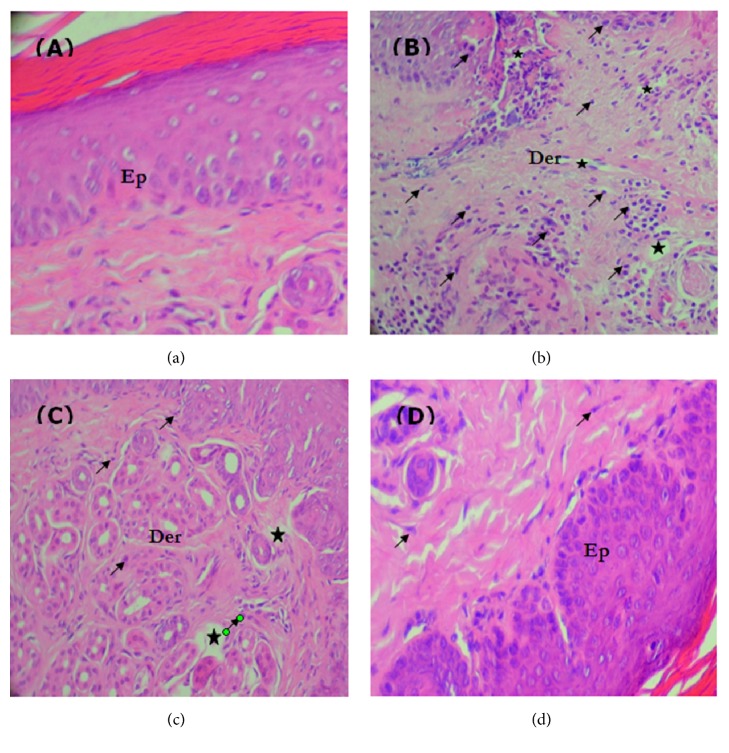
The representative photomicrographs of the skin showing the protective effects of* J. phoenicea *EtOH-H_2_OE against carrageenan-induced inflammation in mice. Controls (a), mice treated with carrageenan (b), the combination of carrageenan and dexamethasone (c), and mice treated with the combination of carrageenan and* J. phoenicea* EtOH-H_2_OE (d). Ep: epidermis, Der: dermis. ★: oedema. ↗: inflammatory cell.

Biopsies from plantar muscles of the untreated group showed normal histological sections (Figure 2). However, biopsies from carrageenan-treated mice (Figure 2(a)) showed numerous inflammatory cells and tissue structure disruption. Biopsies from the* J. phoenicea*-treated mice (Figure 2(d)) showed absence of inflammatory cells.

### 3.6. *In Vivo* Effects on the Malondialdehyde (MDA) Levels

The malondialdehyde levels in oedema paw induced by carrageenan are shown in Table 6. For the carrageenan-treated mice, the dermal MDA levels increased significantly (*P* < 0.001) compared to the normal control group. Nevertheless, the treatment with 100 mg/kg BW of EtOH-H_2_OE showed a significant decrease (*P* < 0.001) compared to the carrageenan group and even the dexamethasone group (10 mg/kg). The treatment with* J. phoenicea* reinstated the MDA formation by 95.37% against only 90.74% recorded in dexamethasone. The malondialdehyde concentrations in the* J. phoenicea* EtOH-H_2_O-treated group were comparable to those of the normal control group.

### 3.7. Effects on Enzymatic Antioxidant Status

The obtained results of SOD, CAT, and GSH levels in the paw oedema tissue of the different tested groups are summarized in Table 6. The carrageenan-induced inflammation mice led to a significant decrease of the dermal SOD, CAT, and GSH levels compared to the normal control mice (*P* < 0.001). However, our results proved that the animals pretreated with* J. phoenicea* EtOH-H_2_OE did not produce any significant differences in enzymatic antioxidant levels compared to the control group. The treatment of inflamed mice with the EtOH-H_2_OE of* J. phoenicea* (100 mg/kg BW) restored the SOD activity by 84.24%, the CAT activity by 91.17%, and the GPx activity by 79.28%, compared to the control group. The results showed that the* J. phoenicea* leaves hydroethanolic extract displayed a protection of 84.24%, 91.17%, and 79.28% for SOD, CAT, and GSH activities, respectively, compared to 78.79%, 61.34%, and 77.85% using dexamethasone as reference drug towards inflammation induced by carrageenan.

### 3.8. Acetic Acid-Induced Writhing Response

Our results showed that the tested extract at different doses and diclofenac sodium used as a standard significantly reduced the writhing number as compared to the control untreated mice (*P* < 0.05). As shown in Table 7, the pain relief was reached in a dose-dependent way with all the tested doses (50, 100, and 150 mg/kg i.p.). The writhing maximum inhibition was 77.33% recorded at 150 mg/kg dose of* J. phoenicea* EtOH-H_2_OE.

The inhibitory effect of* J. phoenicea* EtOH-H_2_OE (77.33%) at the dose of 150 mg/kg was just like that obtained by diclofenac sodium (79.88%) at the dose of 50 mg/kg.

## 4. Discussion

Plants have been commonly used for centuries as potential sources of new anti-inflammatory compounds. Inflammation is a physiological protective response to body tissue injury or bacterial invasion, which involves several intricate factors. During the inflammatory response, the injured tissues release mediators leading to the generation of ROS, which are well known for their deleterious effects [33]. The anti-inflammatory effects of numerous plant extracts may be attributed to the antioxidant property of their phytoconstituents. In the current study, the* J. phoenicea* EtOH-H_2_OE was found to have potent antioxidant activity* in vitro*. The richness of EtOH-H_2_OE of* J. phoenicea* in phenolics known for their powerful antioxidant property and also involved in the modulation of pain and reduction of inflammation [34–36] motivated us to evaluate the analgesic and anti-inflammatory effects of* J. phoenicea* in mice models.

The paw oedema induced by carrageenan is a model often used to assess the anti-inflammatory effects of novel compounds. This inflammation type has two major phases. The first is caused by histamine, leukotriene, kinin, and cyclooxygenase release during the first hour after the carrageenan administration. The second or delayed phase is linked to the generation of prostaglandins, bradykinin, and neutrophil infiltration [37]. In the present investigation, the significant inhibitory activity (*P* < 0.001) shown by EtOH-H_2_OE of* J. phoenicea* over a period of 5 h in carrageenan-induced inflammation was higher than that elicited by dexamethasone. Dexamethasone is a prostaglandin synthesis inhibitor [38]. Our results suggest that the anti-inflammatory effect of EtOH-H_2_OE of* J. phoenicea* may be due to the inhibition of prostaglandin biosynthesis, which is similar to that produced by steroidal anti-inflammatory drugs such as dexamethasone. However, exact mechanism of inhibition of prostaglandin synthesis could be a potential future perspective.

Carrageenan-induced local inflammation was reported to be associated with the generation of reactive oxygen species (ROS) and to play a key role in the genesis of oxidative stress [39]. Previous research has reported that ROS overproduction may lead to increasing lipid peroxidation [40]. A reduction of the MDA level, a biomarker involved in lipid peroxidation, was observed in EtOH-H_2_OE of* J. phoenicea* treated group compared to the untreated group after 5 hours. According to the enzymatic antioxidant analysis,* J. phoenicea* may promote the activities of SOD, CAT, and GSH by boosting a cellular antioxidant protection mechanism. This implies a protective effect of* J. phoenicea* extract by stimulating the expression and the activity of antioxidant enzymes during the inflammatory process.

These results suggest that the extract contains some phytochemical compounds with antioxidant activities which could contribute to the anti-inflammatory process [41, 42]. Boughton-Smith et al. [43] showed that the carrageenan-induced rat paw oedema tissue is sensitive to antioxidants. Therefore, antioxidant components in EtOH-H_2_OE of* J. phoenicea* may also contribute to its anti-inflammatory capacity.

It is well known that following the carrageenan-induced rat paw oedema there are changes in blood parameters that are suggestive of an acute phase reaction leading to a hemostatic imbalance [44]. Carrageenan-induced increase in total white blood cells count and platelets occurs at the site of inflammation due to the release of inflammatory cytokines, which increases the recruitment of neutrophil counts [45]. The reduced count of total white blood cells could be the result of an inhibition of carrageenan-induced total leukocytes, which occurs at the site of inflammation by the phytoconstituents of the hydroethanolic extract of* J. phoenicea*.

It is well established that dexamethasone and other anti-inflammatory drugs inhibit migration of inflammatory cells by inhibiting the release of various chemical mediators [46]. In the present study, the significant decrease in the WBC count caused by the EtOH-H_2_OE of* J. phoenicea* and dexamethasone suggests that these agents are able to reduce inflammation by decreasing the leukocyte migration to the tissue injury sites and possible inhibition of cellular infiltration such as neutrophils and granulocytes.

Platelets have been recognized not only to play a major role in hemostasis but also to participate in inflammation and innate and adaptive immunity responses [47]. It was reported that in the inflamed paw tissue there is platelet aggregation and fibrin deposition [48]. It has been proven that the platelets form aggregates with leukocytes and interact with neutrophils, monocytes, and lymphocytes and therefore play a critical role in the inflammatory process [49]. The decrease in platelet count, observed in EtOH-H_2_OE treated group, suggests a possible inhibitory effect of* J. phoenicea* components on platelet aggregation, which could contribute to the anti-inflammatory process [50].

Most inflammatory biomarkers such as fibrinogen and CRP increased significantly following the inflammation response. However, EtOH-H_2_OE of* J. phoenicea* significantly decreases the mean of fibrinogen and CRP levels. All these findings were also confirmed by histological examination. Indeed, the extract considerably decreases the number of cellular infiltrates and reduces oedema tissue as did reference drug dexamethasone, whereas the paw tissue of untreated mice displayed a subcutaneous oedema with invasive cell infiltration associated with an epidermal ulcer and vascular congestion compared to the treated ones.

The acetic acid-induced writhing protocol was achieved to assess the peripheral analgesic efficiency of test drugs. According to the test, the extract has significantly (*P* < 0.001) decreased the total number of abdominal constrictions by boosting pain inhibitory mediators. The pain sensation is elicited by producing localized inflammatory response due to the release of endogenous substances as well as some other pain mediators such as arachidonic acid via prostaglandin biosynthesis [51]. Prostaglandin products, at damaged tissue sites, contribute to the inflammatory process and pain by increasing the capillary permeability [52]. Our results suggest that* J. phoenicea* contains some phytochemical compounds that can exert anti-inflammatory and antinociceptive effects probably by blocking the release of inflammatory mediators like serotonin, histamine, and prostaglandin and reducing blood flow.

Thus, the anti-inflammatory and analgesic activities are due to the individual or synergistic effect of the* J. phoenicea* components. The richness of* J. phoenicea* extract in terpenoids, polyphenols, and flavonoids may also synergistically promote inhibition of the enzymes involved in the prostaglandin synthesis as previously reported [53, 54].

The GC-MS analysis of* J. phoenicea* EtOH-H_2_OE revealed the presence of hexadecanoic acid in this extract. Earlier reports have evaluated the efficiency of hexadecanoic acid in the anti-inflammatory process and reported that hexadecanoic acid, in studies with isolated Kupffer cells, improved the inhibition of various chemical mediators such as nitric oxide, interleukin-10, tumor necrosis factor-*α*, and prostaglandin E2 [55]. Moreover, recent observations highlighted the anti-inflammatory effects of azelaic acid achieved by reducing reactive oxygen species [56]. On the other hand, the tested extract contained a significant amount of stearic acid. It was reported that these fatty acids fairly produce potent anti-inflammatory and analgesic effects [57]. Our results also suggest that* J. phoenicea* contains promisingly potent phytochemical compounds with anti-inflammatory and antinociceptive properties, which may inhibit the release of inflammatory mediators. EtOH-H_2_OE of* J. phoenicea* may have a potential benefit for the management of pain and inflammatory disorders. These results can justify the traditional use of this plant as a decoction to relieve the pain in Tunisia [16].

## 5. Conclusion

The current study revealed that the hydroethanolic extract obtained from* J. phoenicea* has anti-inflammatory and analgesic activities that were coupled with potent antioxidant capacity attributed to the total extract richness in various chemical compounds. Further studies are required to confirm our results for the use of this extract as a source for new proinflammatory drugs in human beings.

## Figures and Tables

**Table 1:** Phytochemical analysis of *J. phoenicea* EtOH-H_2_OE.

	Terpenoids	Tannins	Alkaloids	Anthraquinones	Saponins	Glycosides
EtOH-H_2_OE	++	++	+	+	++	-

The sign ++ indicates being abundant; the sign + indicates being present; the sign - indicates being absent.

**Table 2:** Amounts of total phenolic compounds and total flavonoids and determined IC_50_ values of the DPPH free radical scavenging assay and *β*-carotene bleaching test of *J. phoenicea* EtOH-H_2_OE. Ascorbic acid and BHT were used as standards.

Extracts	Phenolic content^a^ (mg GAE/g)^b^	Flavonoid content^a^ (mg EQ/g)^c^	DPPH^a^ (IC_50_ *μ*g/ml)	*β*-carotene (IC_50_ *μ*g/ml)
EtOH-H_2_OE	70.3±0.20	11.33±0.05	12.22±0.02	15±0.01
Ascorbic acid	-	-	3.5± 0.20	-
BHT	-	-	-	5.1 ± 0.10

^a^Each value represents the mean ± SD of three experiments.

^b^(mg GAE/g): mg of gallic acid equivalent per g of dry plant extract.

^c^(mg EQ/g): mg of quercetin equivalent per g of dry plant extract.

-: not tested.

**Table 3:** GC/MS analysis of *J. phoenicea* EtOH-H_2_OE.

Compounds	t_*R*_ (min)	Content (%)	Characteristic mass fragments
***Phenolic compounds***			
Gallic acid	31.801	0.71	458, 281, 443, 355, 399, 179, 147, 73
Caffeic acid	28.571	1.65	219, 381, 396, 73
*ρ*-coumaric acid	30.937	0.55	219, 249, 293, 308, 73

***Alcohols***			
Resorcinol	12.690	0.68	257, 239, 209, 147, 112, 91, 73
Threitol	14.726	0.16	307, 217, 189, 147, 103, 73
Erythritol	14.909	0.89	307, 217, 189, 147, 103, 73
Xylitol	23.410	1.42	307, 217, 191, 147, 103, 73
Inositol	27.978	0.66	147, 191, 205, 217, 265, 306, 318, 73

***Sugars***			
L-Fructose	18.636	0.23	204, 147, 73
*α*-D-Galactopyranoside	29.048	0.17	204, 175, 147, 103, 73
*β*-D-Glucopyranose	33.148	1.22	117, 147, 204, 246, 73
Galactopyranose	39.972	0.15	103, 147, 204, 249, 307, 331, 73

***Fatty acids***			
Lauric acid	5.771	0.42	95, 117, 145, 210, 229, 257
Suberic acid	6.218	0.43	95, 129, 149, 187, 217, 259, 303, 73
Hexadecanoic acid	12.310	7.41	117, 145, 129, 132, 313, 73
Azelaic acid	7.323	2.45	97, 117, 147, 171, 201, 243, 273, 317, 73
Stearic acid	20.884	5.69	117,147, 201, 297,341, 423, 73
Oxiraneoctanoic acid	22.875	2.02	87, 128, 177, 199

***Others***			
Ethylamine	6.518	0.46	174, 100, 86, 73
Propylene glycol	7.206	3.11	147, 117, 73
2-Ethylhexanol	8.568	0.61	187, 103, 75
Eseroline, 7-bromo-methylcarbamate	10.479	3.15	99, 130, 160, 188, 217, 240, 266, 298, 353
Xylonic acid, 1, 4 lactone	18.475	0.14	364, 321, 246, 217, 189, 147, 189, 147, 117, 73
Mannonic acid 1, 4 lactone	29.831	0.7	451, 361, 319, 220, 189, 147, 103, 73
D-Gluconic acid	33.477	0.6	219, 381, 396, 73

**Table 4:** Effects of *J. phoenicea* EtOH-H_2_OE and dexamethasone on paw oedema tissue after carrageenan administration. Values represent mean ± SD (*n* = 6) in each group. Each value represents the mean ± SEM of results from six animals.

	**1 h**	**2 h**	**3 h**	**4 h**	**5 h**
**Control**	0.15± 0.03^a^	0.1± 0.01^a^	0.17± 0.05^a^	0.18± 0.04^a^	0.2 ±0.06^a^

**Carr**	0.75±0.104^b^	1.2 ±0.12^c^	1.42± 0.104^c^	1.38 ±0.089^d^	1.36± 0.089^d^

**Carr + DEX**	0.7± 0.2^b^	0.93± 0.18^b^	1.2± 0.14^bc^	0.58± 0.1^c^	0.56± 0.09^c^

**Carr + *J. phoenicea* EtOH-H**_**2**_ **OE**	0.68 ±0.103^b^	0.99± 0.136^bc^	1.1 ±0.08^b^	0.36±0.25^b^	0.27 ±0.26^b^

^a,b,c,d^Different letters in the same column indicate significant differences (a > b > c > d; *P* < 0.05).

**Table 5:** White blood cells and platelets count and levels of fibrinogen and C-reactive protein (CRP). Values represent mean ± SD (*n* = 6) in each group.

	**WBC**	**PLT**	**Fibrinogen (g/l)**	**CRP (mg/ml)**
**Control**	3.68±0.4 ^a^	292±4.2 ^a^	3.35±0.05 ^a^	8±0.83 ^a^

**Carr**	12.13±0.95 ^c^	1207±11.3 ^b^	5.64±0.07 ^b^	24±0.94 ^b^

**Carr + DEX**	8.03±0.62 ^b^	965±7.9 ^b^	3.53±0.01 ^b^	12±0.09 ^a^

**Carr + *J. phoenicea* EtOH-H**_**2**_ **OE**	4.4±0.31 ^a^	779.47±6.04 ^c^	3.48±0.03 ^c^	9±0.36 ^a^

^a,b,c^Different letters in the same column indicate significant differences (a > b > c; *P* < 0.05).

**Table 6:** Effects of *J. phoenicea* EtOH-H_2_OE and dexamethasone on CAT, SOD, GPx, and MDA activities in carrageenan-induced paw oedema.

**Treatment**	**MDA** **(nmol /mg protein)**	**SOD** **(units/mg protein)**	**CAT** **(**μ**mol H**_**2**_**O**_**2**_**/min/mg protein)**	**GPx** **(**μ**mol GSH/mg protein)**
**Control**	1.08 ± 0.07 ###	8.44 ± 0.31###	186.04± 6.13###	5.6± 1.07 ###
**Carr**	1.94± 0.01*∗∗∗*	2.26 ± 0.12*∗∗∗*	77.12± 1.6*∗∗∗*	1.23± 0.07*∗∗∗*
**Carr + DEX**	0.98 ± 0.04*∗* ###	6.65 ± 0.57*∗∗∗* ###	114.13± 3.9	4.36 ± 0.19*∗∗* ###
**Carr + *J. phoenicea* EtOH-H**_**2**_ **OE**	1.03 ± 0.009 ###	7.11 ± 0.13*∗∗∗* ###	169.62± 4.3##	4.44 ± 0.31*∗* ###

Values are mean ± SE for six rats in each group. *∗∗∗* represents *P* < 0.001; *∗∗* represents *P* < 0.01;*∗* represents *P* < 0.05.

SOD: superoxide dismutase, CAT: catalase, GSH: glutathione peroxidase, MDA: malondialdehyde.

*∗∗∗* (*P* < 0.001) represents highly significant difference in comparison with control mice.

*∗∗* (*P* < 0.01) represents moderately significant difference in comparison with control mice.

*∗* (*P* <0.05) represents significant difference in comparison with control mice.

### (*P* <0.001) represents highly significant difference in comparison with Carr group.

## (*P* < 0.01) represents moderately significant difference in comparison with Carr group.

**Table 7:** Effects of EtOH-H_2_OE of *J. phoenicea* on acetic acid-induced writhing in mice.

**Group**	**Dose (mg/kg)**	**Percentage of inhibition (**%**)**
**Acetic acid**	(10ml/kg)	0 ^f^
**Diclofenac sodium**	50	79.88 ^b^
**Diclofenac sodium**	100	100 ^a^
***J. phoenicea* EtOH-H**_**2**_ **OE **	50	20.15 ^e^
***J. phoenicea* EtOH-H**_**2**_ **OE **	100	42.79 ^d^
***J. phoenicea* EtOH-H**_**2**_ **OE **	150	77.33 ^c^

Values are expressed as mean ± SD (*n* = 6).

^a,b,c,d,e,f^Different letters in the same column indicate significant differences (a > b > c > d > e > f; *P* < 0.05).

## Data Availability

No data were used to support this study.
